# University of Louisville

**Published:** 1850-10

**Authors:** 


### THE WESTERN JOURNAL.

Third Series.—Vol. VI.—No. IV.

LOUISVILLE, OCTOBER 1, 1850.

UNIVERSITY OF LOUISVILLE.

It affords us sincere pleasure to state, that Dr. Drake returns to the chair in the University of Louisville of which he was so long the incumbent. Our readers, we are sure, will be gratified by this announcement. The institution is fortunate in having secured the services of a teacher so widely and favorably known. Dr. Drake's work, which has been noticed in our Journal, will strengthen the desire of students to hear his lectures on the Theory and Practice of Medicine. It is a compliment due to the author of this great work, that he should be recalled to the school in which he has spent some of the best years of his life. He will be cordially welcomed back by his old colleagues, and by his former pupils, many of whom have returned, and will be in the College hall to receive him on the first day of November.

We are also gratified in being able to announce, that Dr. Paul F. Eve takes the chair of Surgery in the above institution. Dr. Eve, too, has a wide reputation. He has been for a number of years connected with the Medical College of Georgia, and is distinguished as a teacher and a surgeon. We have had more than one occasion to notice the operations of Dr. Eve, and have repeatedly alluded to the *Southern Medical and Surgical Journal,* which he edited for several years with marked ability.

The friends of the University cannot but be gratified in seeing the places of Bartlett and Gross filled by successors so worthy as Drake and Eve. Y.

